# Removal of Cr(VI) from Aqueous Solutions Using Biowastes: Tella Residue and Pea (*Pisum sativum*) Seed Shell

**DOI:** 10.1155/2022/7554133

**Published:** 2022-01-28

**Authors:** Abayneh Kebede, Kassim Kedir, Fekadu Melak, Tsegaye Girma Asere

**Affiliations:** Chemistry, College of Natural Sciences, Jimma University, P. O. Box 378, Jimma, Ethiopia

## Abstract

The wide use of chromium (Cr) in different industries led to the release of a considerable amount of Cr(VI) into water bodies. Exposure to Cr(VI) can cause diseases in humans and animals. Therefore, low-cost technology for Cr(VI) removal is required. In this study, the biowastes, “Tella” residue (TR) and Pea (*Pisum sativum*) seed shell (PSS), were evaluated for their Cr(VI) removal efficiency from aqueous solutions. The physicochemical properties of adsorbents were studied, and the adsorbents were further characterized using FTIR and XRD. Batch adsorption experiments have shown that the Cr(VI) uptake was pH-dependent and found to be effective in a wide range of pH values (pH 1 to 10) for PSS. The kinetics of Cr(VI) removal by the adsorbents was well expressed by the pseudo-second-order model. The experimental equilibrium adsorption data fitted well with Freundlich isotherm indicating multilayers adsorption. The estimated Cr(VI) adsorption capacities of TR and PSS were 15.6 mg/g and 8.5 mg/g, respectively. On top of this, the possibility of reusing adsorbents indicates the potential applicability of TR and PSS for the treatment of Cr(VI) contaminated water. Further study on the evaluation of the efficiency of the adsorbents using real chromium-contaminated wastewater is recommended.

## 1. Introduction

Chromium- (Cr-) containing compounds are widely used in industries, such as tanneries, metal plating, textiles, batteries, pesticides, and fertilizers, that can result in huge quantities of Cr discharged into the environment [1]. For instance, tannery industries consume only about 60 to 70% of the total Cr employed in the tanning process, and the remaining 30 to 40% is released into the environment as tannery effluents [2]. Chromium occurs in the water environment in two relatively stable oxidation states, Cr(III) and Cr(VI). Most tanneries use Cr(III); however, the redox conditions of the environment convert it to Cr(VI) species. Cr(III) is essential for the proper functioning of living organisms, playing an important role in the metabolic processes of certain enzymes and stimulating the synthesis of cholesterol and fatty acids [3]. In contrast, Cr(VI) is highly toxic, causing severe physiological and neurological effects in humans and animals [4]. This could be because Cr(VI) is water-soluble, is mobile, and can exist in a variety of forms in aqueous systems, including CrO_4_^2-^, HCrO_4_^−2^, and Cr_2_O_7_^2-^ depending on the pH and redox potential of the medium [3, 5]. Thus, the WHO has established a maximum permissible level of Cr(VI) in drinking water of 0.05 mg/L [6], whereas the US EPA has established a limit of 0.1 mg/L for Cr(VI) discharged into surface water [7]. This leads to the search for efficient removal of Cr(VI) from water/wastewaters.

Chemical precipitation (primarily by reducing Cr(VI) to Cr(III)) [8], ion exchange [9], electrodialysis [10], membrane separation [11], and adsorption [12] are common methods for removing Cr(VI) from water/wastewaters. A large amount of sludge, high cost, and the need for skilled manpower are among the challenges encountered for most of the methods of Cr(VI) removal. Adsorption has been given due attention these days because of its high removal efficiency, easier applicability in rural areas, and low cost, compared to the alternative technologies listed above [13]. Among the few adsorbents with high adsorption capacity for Cr(VI) removal are chitosan, modified chitosan, steel industry waste material, rice husk, activated alumina, and neem bark [14–16]. Biosorption is among the prominent processes for removing metal ions from water and wastewater by using nonliving biomass [17–19]. Biowastes are abundant and are mainly composed of cellulose and lignin. The common functional groups in biowastes are hydroxyl (-OH), carboxyl (-COOH), phenolic, amino (-NH_2_), sulphydryl (-SH), alcoholic, and ester groups, which have the potential to remove metal ions from water [13]. Glycine-modified chitosan [20], almond green hull waste material [21], biochar from walnut shell [22], rice husk [23], and so on have been considered as efficient and green processes [24] to remove Cr(VI) and other heavy metals from water. Even though biosorption is an eco-friendly alternative to conventional methods, there are only a few commercialized adsorbents for Cr(VI) removal from water. Further, challenges to adsorbents such as limitation of applicability, inefficiency, and cultural taboo should be solved with alternatives.

Accordingly, the objective of this work was to assess the Cr(VI) adsorption potential of selected biowastes locally available in Ethiopia. Batch adsorption experiment was performed for removal of Cr(VI) from aqueous solutions using selected locally available biomaterials: charcoal of acacia tree, eucalyptus tree ash, avocado (*Persea americana*) seed shell, mango (*Mangifera indica* L.) seed shell, peas (*Pisum sativum*) seed coat, “korch” (*Erythrina brucei* Schweinf) bark, “Tella” residue (local alcoholic beverage residue), eggshell, and petiole of false banana (the midrib, which divides the blade into two lamina halves), and barley hull (*Hordeum vulgar*e L. (Poaceae) residue).

“Tella” is a traditionally fermented beverage commonly consumed in Ethiopia, a beer brewed at home. It is made from different ingredients such as barley or wheat, hops (hop or “Gesho”), or spices, which are abundantly available in the localities and served for many purposes [25]. “Tella” also plays important role in food security and income generation for the local community [26]. It has been prepared with four main phases: Phase I, a stage in which a small volume of water, malt, and “Gesho” (*Rhamnus prinoides*) is added, the “Tensis” stage; Phase II, a stage of additional starch source called “Enkuro,” which is added on the second day of “Tensis”; Phase III, addition of well-cooked barely called “Asharo/Derekot”; and Phase IV, drinkable “Tella” production by adding extra water and waiting for 3–5 h. Finally, the “Tella” will be separated from the residue by decantation and filtration. Normally, the biowaste, Tella residue (TR), is served for cattle or discarded. In this study, it was employed for the adsorptive removal of Cr(VI).

## 2. Materials and Methods

### 2.1. Reagents

A 1000 mg/L stock solution of Cr(VI) was prepared from K_2_Cr_2_O_7_ (99.5%, FINKEM, England) using 1 L of distilled water. Calibration standards were prepared by diluting the stock solution with distilled water. Solutions of 0.1 M HCl (37%, Riedel-de Haën, Germany) and 0.1 M NaOH (90%, BDH, England) were used to adjust the pH of working solutions. Different concentrations of Cr(VI) were freshly prepared from the stock solution and directly used for desired experiments. During residual Cr(VI) determination, the chelating agent was prepared by dissolving 0.25 g of 1,5-diphenylcarbazide (98%, Analar, England) in 50 mL of acetone (BDH Chemicals Ltd.) in a volumetric flask. Then, it was transferred into a brown bottle and used immediately. H_2_SO_4_ (98%, UNI CHEM, Germany) and HNO_3_ (69%, LOBA, Chem, India) were also used during the experiment. All experiments were performed in triplicate with a blank and a control, and the average results were reported.

### 2.2. Adsorbent Preparation

**Figure 1:**
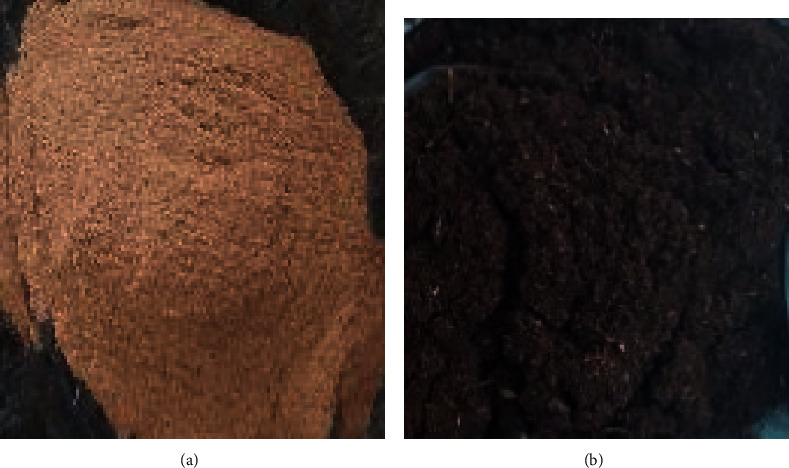
Photograph of adsorbents: (a) PSS and (b) TR.

Locally, available adsorbents, such as avocado (Persea americana) seed, mango (*Mangifera indica* L.) seed shell, eggshell, *Ensete ventricosum* midrib leaf, barely (*Hordeum vulgare* L. (Poaceae) bran), coffee (*Coffea arabica* L) residue, “Tella” residue, Eucalyptus tree ash, Acacia tree charcoal, pea (*Pisum sativum*) seed shell, and korch (*Erythrina abyssinica*) were collected from different places of Jimma city, Ethiopia. All these adsorbents were washed with tap water repeatedly, rinsed with distilled water to remove water-soluble impurities, and dried in the air. After drying, all the adsorbents were ground, sieved to a mesh size of 300 µm, and stored in airtight containers. “Tella” residue (TR) was collected from the Kochi area (Jimma City) and air-dried (Figure 1), and the pea (*Pisum sativum*) seed shell (PSS) was collected from the Milling House, Ajip area.

### 2.3. Characterization of Adsorbents

#### 2.3.1. Physicochemical Characteristics

The pH of the adsorbents was measured using a pH meter in a 1 : 10 adsorbent/water ratio [27]. The point of zero charge (pHpzc) of each adsorbent was measured by using 0.01 M and 0.1 M NaCl solutions as an electrolyte and adding 0.1 M NaOH or HCl solutions [27]. The surface area of adsorbents was calculated by using Sear's technique [28]. The moisture and ash content of the samples were determined by using the ASTM D2867-91 technique [29] and the ASTM D2866-94 method [30], respectively. The volatile matter was determined following the method employed by Zulkania et al. [31]. The fixed carbon of samples was calculated by subtracting the total of moisture content, ash content (percent), and volatile matter (percent) from 100 [31].

#### 2.3.2. X-Ray Diffraction (XRD)

The crystallographic structure of the powdered TR and PSS samples was investigated with X-ray diffraction (D8 ECO XRD). The XRD patterns were obtained using a diffractometer (CuK radiation, *l* = 1.5418) set to 40 kV, 25 mA, and 2q ranges of 10° to 90°.

#### 2.3.3. Fourier Transform Infrared (FTIR) Spectral Analysis

FTIR characterization of the adsorbents was performed to identify the existing functional groups that might be involved in the Cr(VI) uptake process. The adsorbents were characterized using an FTIR spectrophotometer (Perkin Elmer Spectrum 2.0) before and after the adsorption process in wavenumbers from 4000 to 400 cm^−1^. Each adsorbent powder was mixed with KBr particles and made to the required pellet thickness for infrared analysis.

### 2.4. Batch Adsorption Studies

Batch adsorption experiments were carried out at room temperature with a horizontal shaker using a thermostatic water bath (GrantGLS400, England) at a speed of 200 rpm. In the experiment, Cr(VI) solutions of known initial concentrations and the desired pH were agitated to equilibrium and filtered. The filtrate was analyzed for residual Cr(VI) concentration using a double beam UV-VIS spectrophotometer (Analytic Jena Specord200 Plus, Germany). The solution pH was measured using a digital pH meter (HANNA instruments, pH 211).

Equations (1) and (2) were used to calculate the Cr(VI) removal efficiency (%) and the amount of Cr(VI) adsorbed per gram of adsorbent (mg/g), respectively:

(1) $$\begin{array}{c} \text{Cr}\left(\text{VI}\right)\text{ removal }\left(\%\right)=\left(\frac{C_{0}\;-C_{e}}{C_{0}}\right)\times 100, \end{array}$$

(2) $$\begin{array}{c} q_{e}=\left(C_{0}-C_{e}\right)\frac{V}{m}, \end{array}$$

where *C*_0_, and *C*_e_ are the initial Cr(VI) concentration in solution before mixing with the adsorbent (mg/L) and the concentration of Cr(VI) at equilibrium (mg/L), respectively; *V* is the volume of solution (*L*) and *m* (g) is the mass of adsorbent used.

#### 2.4.1. Kinetics

To explore the adsorption kinetics of Cr(VI) ions onto the TR and PSS, the experimental kinetic data of Cr(VI) adsorptions were fitted into nonlinear pseudo-first-order (PFO) (see (3)) and pseudo-second-order (PSO) (see (4)) models:

(3) $$\begin{array}{c} q_{t}=q_{e\;}\left(1-\;\exp^{-K_{1}\;t}\right), \end{array}$$

(4) $$\begin{array}{c} q_{t}=\frac{K_{2}q_{e}^{2}t}{1\;+\;K_{2}q_{e}t}, \end{array}$$

where *q*_*e*_ is the Cr(VI) uptake in mg/g at equilibrium, *q*_*t*_ is the uptake at any time *t* (mg/g), and *k*_1_ and *k*_2_ are the PFO (L/min) and PSO (g/mg min) rate constants, respectively.

#### 2.4.2. Isotherms

Adsorption isotherms are useful to describe the adsorption behavior of a solute on the adsorbent at equilibrium at a constant temperature. As a result, the equilibrium experimental data of Cr(VI) adsorption on TR and PSS were fitted to two common isotherm models: the Langmuir and Freundlich isotherms, as shown in (5) and (6), respectively:

(5) $$\begin{array}{c} q_{e}=\frac{Q_{\max}bC_{e}}{\left(1+bC_{e}\right)}, \end{array}$$

(6) $$\begin{array}{c} q_{e}=K_{F}C_{e}^{1/n}, \end{array}$$

where *Ce* (mg/L) is the concentration of Cr(VI) in the aqueous phase at equilibrium, *Q*_max_ (mg/g) is the maximum adsorption capacity based on the Langmuir equation, *b* (L/mg) is the Langmuir constant, *K*_*F*_ (mg^1−1/*n*^*L*^1/*n*^/g) is the adsorption coefficient based on the Freundlich equation, and ^1^/*n* is the adsorption intensity based on the Freundlich equation.

## 3. Results and Discussion

### 3.1. Adsorbent Characterization

Table 1 shows the physicochemical characteristics of TR and PSS. The adsorbents are characterized by their relatively low ash, volatile matter, and moisture contents, as well as their high content of fixed carbon. The values given in Table 1 are comparable to some of the (activated) biowastes [32]. Therefore, the selected biowaste materials have good quality of adsorbents and are suitable for the removal of Cr(VI) from aqueous solutions.

**Figure 2:**
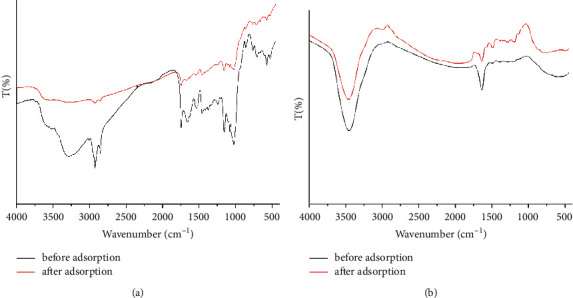
FTIR analysis of adsorbent surfaces (a) TR and (b) PSS before and after adsorption.

Figure 2 shows the FTIR spectra before and after the adsorption of Cr(VI) for TR and PSS, in which the absorption bands appeared in the wavenumber range of 4000 cm^−1^ to 400 cm^−1^. After Cr(VI) adsorption, the major absorption bands of TR and PSS show a change in intensity and a shift in absorption wave number, indicating interaction of Cr(VI) ions with the functional groups present on the adsorbent surfaces [21].

The broad absorption band at about 3275 cm^−1^ is due to the stretching of the hydroxyl functional group (−OH) of the carboxylic group present in TR (Figure 2(a)). The bands near 2922 cm^−1^ and 2853 cm^−1^ are attributed to the (C–H) asymmetric and symmetric stretching vibrations, respectively. The bands at about 1745 cm^−1^ could be due to the carbonyl C=O functional group, and the bands at 1023 cm^−1^ may be due to the C−O bond stretching of the adsorbent (TR). In the FTIR spectra of PSS (Figure 2(b)), a medium band at about 3450 cm^−1^ may be assigned to be the stretching of the N−H group of an amide bond. The absorption band that appeared at 1635 cm^−1^ could be due to the C=O group of the amide bond in the PSS [33].

**Figure 3:**
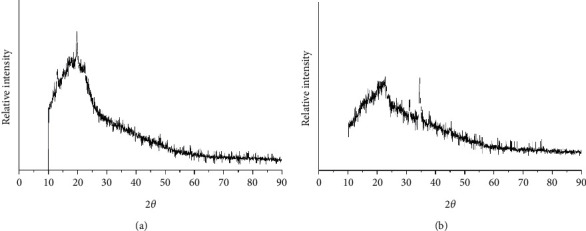
XRD data of (a) TR and (b) PSS and after adsorption.

The XRD pattern for both adsorbents (TR and PSS) was shown in Figures 3(a) and 3(b), respectively. The whole base of XRD diffractogram peaks for both adsorbents were found to be almost as wide and less intense, indicating the amorphous nature of the adsorbent materials. The characteristic diffraction peak of TR was observed at 20°, whereas for PSS, a sharp peak at 35° was observed in addition to the broad peak at 20°.

### 3.2. Screening of Biowaste Materials

**Figure 4:**
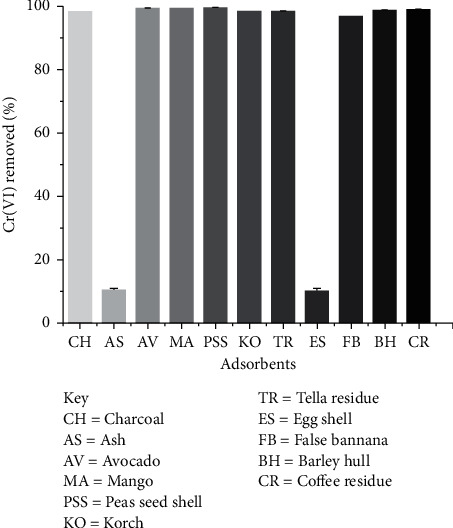
Screening of adsorbents for Cr(VI) removal (conditions: initial concentration of Cr(VI) 5 mg/L, adsorbent dose 2.5 g/L, pH 2, contact time 24 h, shaking speed 200 rpm, and temperature 25°C).

Screening of biowaste materials for their Cr(VI) removal efficiency was evaluated and results were presented in Figure 4. The Cr(VI) removal efficiency of all the tested adsorbents was ≥97% except for ash and eggshell, in which only about 10% of Cr(VI) were removed. Hence, TR and PSS were selected for further investigation for the removal of Cr(VI) based on their high Cr(VI) removal efficiency and also it has not been reported so far in the area.

### 3.3. Effect of Contact Time

**Figure 5:**
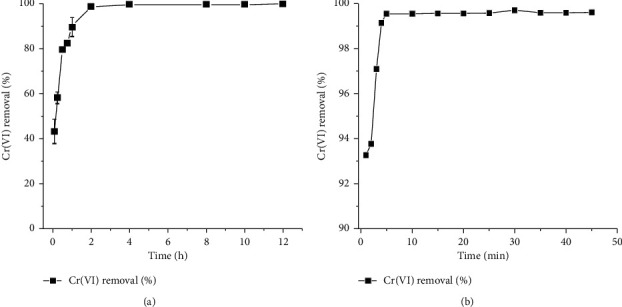
Effect of contact time on Cr(VI) removal by (a) TR and (b) PSS (conditions: initial concentration of Cr(VI) 5 mg/L, adsorbent dosage 2.5 g/L, shaking speed 200 rpm, temperature 25°C, and pH 2).

The effect of contact time is one of the important parameters that influence the adsorption of Cr(VI). The time-dependent Cr(VI) adsorption on TR and PSS adsorbents was studied by varying the contact time from 5 min to 12 h and 1 min to 1 h, respectively. Figures 5(a) and 5(b) show the effect of contact time on Cr(VI) adsorption efficiency.

As indicated in Figure 5, Cr(VI) removal efficiency increases with increasing contact time. Approximately 43% and 99% of Cr(VI) were removed in the first 5 min by TR and PSS adsorbents, respectively. The rapid kinetics of adsorption rate, particularly by PSS, is due to the abundance of active sites on the adsorbent's external surface, which are available for interaction with Cr(VI). At later stages, the rate of adsorption gets slightly slower. These are commonly observed phenomena in adsorption processes as reported in literature [34, 35]. The adsorption process reached equilibrium after agitating the mixture for 2 h for TR and 10 min for PSS adsorbents. However, a contact time of 4 h for TR and 1 h for PSS has been chosen for subsequent adsorption experiments to ensure that maximum adsorption of Cr(VI) occurs.

#### 3.3.1. Adsorption Kinetics

**Figure 6:**
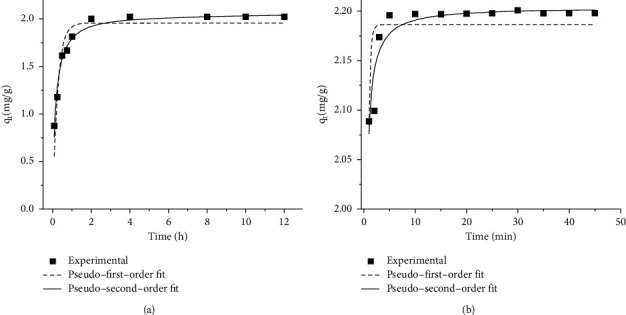
PSO and PFO plot for adsorption of Cr(VI) onto (a) TR and (b) PSS.

The experimental data were evaluated using the nonlinear form of pseudo-first-order (PFO) and pseudo-second-order (PSO) kinetics, as shown in Figure 6. The correlation coefficient (*R*^2^) and chi square (*χ*^2^) values were compared to determine the better-fitted model as described in Table 2. The PSO model fits the experimental data better for both adsorbents than the PFO model as it has a high correlation coefficient and low *χ*^2^ values for both kinds of adsorbents Hence, it can be concluded that the adsorption of Cr(VI) on TR and PSS follows pseudo-second-order kinetics. Furthermore, the initial adsorption rates (V0, mg/g min) were calculated based on *V*0 = *k*2*qe*2, and the Cr(VI) adsorption rate of PSS (35.76 mg/g min) was higher than that of TR (14.26 mg/g min). This may be due to the difference in Cr(VI) affinity for –NH_2_, the active functional group in PSS, and the –COOH group in TR. A high initial slope for both TR and PSS is advantageous for real applications because it allows for a high sorption capacity with low Cr(VI) residual concentrations in a short period of time and a low Cr(VI) residual concentration in the effluent [36].

**Figure 7:**
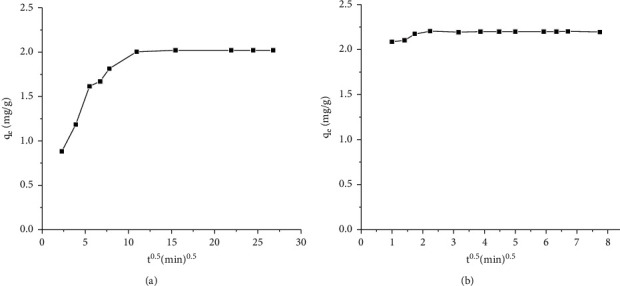
Intraparticle diffusion plots of Cr(VI) adsorption on (a) TR and (b) PSS (initial concentration of Cr(VI) 5 mg/L, adsorbent dosage 2.5 g/L, shaking speed 200 rpm, temperature 25°C, and pH 2).

The Weber and Morris [37] model (see (7)) was also employed to determine if pore diffusion or internal diffusion is the rate-determining step in the adsorption process. Pore or internal diffusion was characterized using the relationship between specific sorption (*q*_*t*_) and time (*t*^0.5^) as

(7) $$\begin{array}{c} q_{t}=k_{p}t^{0.5}+c\;, \end{array}$$

where *q*_*t*_ is the amount of Cr(VI) adsorbed on TR or PSS (mg/g) at time *t* and *k*_*p*_ (mg/(g·min^0.5^) is the intraparticle diffusion rate constant. If the rate-limiting step is pore or intraparticle diffusion, the plot of *q*_*t*_ versus *t*^0.5^ should be a straight line and pass through the origin. Each curve shows a multilinear plot (Figures 7(a) and 7(b)). However, each curve does not pass through the origin indicating that intraparticle diffusion was not the only rate-determining step. Therefore, the adsorption of Cr(VI) and its kinetics could be the overall effect of the external diffusion transport of Cr(VI), the intraparticle diffusion of the ions, and the adsorption of Cr(VI) ions by the active sites on the TR and PSS adsorbents [38]. Adsorption of Cr(VI) onto activated carbon of bamboo waste [39] and nutshell powder [40] yielded similar results.

### 3.4. Effect of pH

**Figure 8:**
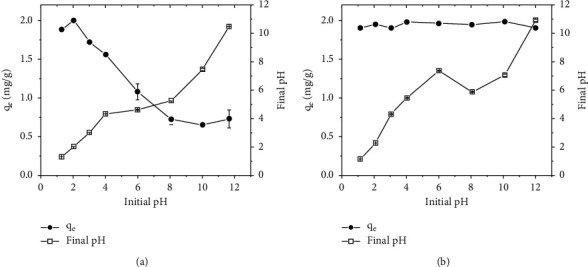
Effect of pH on the absorption of Cr(VI) onto TR (a) and PSS (b) (initial concentration of Cr(VI) 5 mg/L, shaking speed 200 rpm, adsorbent dose 2.5 g/L, and temperature 25°C.

The effect of pH on Cr(VI) adsorption was studied for TR and PSS adsorbents at fixed mass using 5 mg/L Cr(VI) solution, shaking for 4 h and 1 h contact times, respectively (Figure 8(a) and 8(b)). The results demonstrated that maximum Cr(VI) adsorption occurred at pH 2 for TR and significantly decreased by increasing the pH values up to 12. A similar observation was reported for several biowaste materials: compost [41], neem sawdust, mango sawdust, wheat shell, sugarcane bagasse, and orange peel [42]. PSS, on the other hand, demonstrated remarkably high Cr(VI) adsorption efficiency over a wide pH range of 1 to 10, potentially avoiding the need for pH adjustment in real-world applications. However, pH 2 was selected for both TR and PSS in further optimization of other adsorption parameters, which is in line with the actual pH of most industrial effluents, such as tannery, electroplating, and chromium plating effluents [28]. High Cr(VI) removal (>99%) in a wide pH range (pH 2 to 10) was also observed in a previous study using almond green hull [21] at an adsorbent dose of 8 g/L and an initial Cr(VI) concentration of 20 mg/L.

In an acidic environment, among the most predominant Cr(VI) forms are hydrogen chromate (HCrO4-) at pH < 6 and chromic acid (H_2_CrO_4_) at pH < 1, whereas in an alkaline medium (pH > 7), chromate CrO_4_^2−^) is the major form of Cr(VI) [3]. The point of zero charge (pH_PZC_) is considered an important parameter when studying the effect of pH on the adsorption capacity of adsorbents. The experimental results showed that pHPZC of TR and PSS was found to be 4.5 and 6.0, respectively. When the pH < pHPZC, the surface of the adsorbent is positively charged, enhancing the adsorption of the negatively charged Cr(VI) species (HCrO_4_^−^) through electrostatic forces of attraction [43, 44]. However, when the pH of the solution gets very low (pH ≤ 1), the adsorption decreases due to the decline in the electrostatic force of attraction between the positively charged adsorbent surface and the predominant neutral species of chromium (H_2_CrO_4_). When the pH of the solution increases (pH > pH_PZC_), the surface of the adsorbent acquires a net negative charge, resulting in a decrease in the adsorption of Cr(VI) through electrostatic repulsions [45].

### 3.5. Effect of Initial Concentration

**Figure 9:**
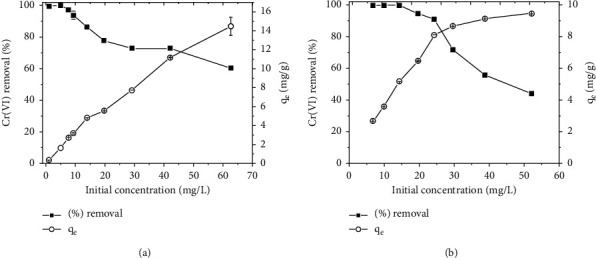
Effect of initial concentrations on adsorption of Cr(VI) onto TR (a), PSS (b) at optimum pH, shaking speed 200 rpm, adsorbent dose 2.5 g/L, and temperature 25°C.

The effect of the initial concentration of Cr(VI) on the adsorption process was investigated by varying the Cr(VI) concentration from 1 mg/L to 80 mg/L for both TR and PSS adsorbents. The results are shown in Figure 9.

As shown in Figure 9, the percent removal of Cr(VI) from the solution decreased with an increase in the initial Cr(VI) concentration from 1 mg/L to 80 mg/L. At lower Cr(VI) concentrations, the adsorbents' available active sites were sufficient to accommodate the few available Cr(VI) ions. However, as Cr(VI) concentrations increase, the available sites of adsorption to an initial number of Cr(VI) ions decrease and removal efficiency decreases for both TR and PSS adsorbents [28]. However, as the initial Cr(VI) increased, the adsorption capacity of the adsorbents increased from 2.7 to 8.5 mg/g for TR and PSS, respectively, and from 5.5 to 15.6 mg/g for TR and PSS. This could be due to the presence of more Cr(VI) per unit mass of the adsorbents, which increases the mass transfer of Cr(VI) ions from the bulk solution to the adsorbent surface.

### 3.6. Adsorption Isotherm

**Figure 10:**
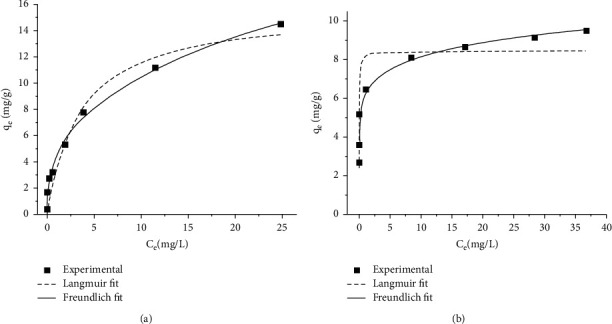
Langmuir and Freundlich isotherm model for the adsorption of Cr(VI) onto (a) TR and (b) PSS.

The experimental data obtained for the adsorption of Cr(VI) on the adsorbents (TR and PSS) are plotted with Langmuir and Freundlich isotherm models (Figure 10), and the isotherm constants are presented in Table 3.

The high correlation coefficient (R2) 0.9507 for the Langmuir model and the Freundlich model with *R*^2^ = 0.9885 (Table 3), indicating the Cr(VI) adsorption onto TR can be modeled using either of the models. However, the Freundlich model gave the highest correlation coefficient values (R2) 0.9885 for TR and 0.9529 for PSS and lower chi-square values for TR and PSS, *χ*2 = 0.3283 and 0.90006, respectively (Table 3). On top of that, the examination of the distribution of residuals (experimental adsorbed amount) of Cr(VI) minus the theoretical fitted Cr(VI) amount were tested for the isotherms. The better fit of the Freundlich model suggests that the removal of Cr(VI) by TR and PSS probably occurs through physical adsorption as well as a heterogeneous distribution of active sites on the surface of adsorbents [46]. The value of *q*_max_ (obtained from Langmuir plots) for TR (15.61 mg/g) was nearly twice that for PSS (8.45 mg/g). TR's higher Cr(VI) adsorption capacity compared to PSS can be attributed to the involvement of more functional groups in the adsorption process. This could be witnessed by a large decrease in the intensity of the major FTIR bands of TR after Cr(VI) compared to PSS (Figure 2). The suitability of the TR and PSS adsorbents for Cr(VI) ions was predicted using the separation factors (*R*_L_), which were calculated using the equation *R*_L_ = 1/(1 + bC0). The adsorption process is irreversible if *R*_L_ = 0, favorable if 0 < RL < 1, linear if RL = 1, and unfavorable if *R*_L_ > 1. In this study, the obtained *R*_L_ values were in the ranges (0 < RL < 1) (Table 3), indicating that Cr(VI) adsorbs well to TR and PSS adsorbents.

Table 4 summarizes the maximum adsorption capacities of some biowaste adsorbents in comparison to the TR and PSS adsorbents. When compared to several other adsorbents, this evaluation revealed a generally good maximum adsorption capacity of Cr(VI) onto the TR and PSS adsorbents (Table 4). Hence, both TR and PSS can be considered to be viable adsorbents for the removal of Cr(VI).

### 3.7. Cr(VI) Adsorption Cycles

**Figure 11:**
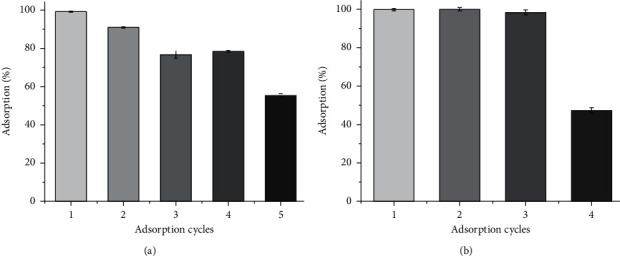
Cr(VI) adsorption cycle of (a) TR (b) PSS.

The recycling of any adsorbent is of great importance as a cost-effective process in water treatment [52]. The effect of recycling frequency on the Cr(VI) adsorption performance of TR and PSS was evaluated (Figures 11(a) and 11(b)). The adsorption efficiency of Cr(VI) ions was decreased from nearly 100% (the 1^st^ cycle) to about 80% (the 4^th^ cycle) for TR, but it works well until the 3^rd^ cycle for PSS, indicating that these adsorbents are efficient for removal of Cr(VI) ions from water/wastewater.

## 4. Conclusion

Low-cost biowastes were screened for the removal of Cr(VI) from aqueous solutions. It was found that TR and PSS were found very effective among the evaluated biowastes for wide concentration levels of Cr(VI). The effective removal of Cr(VI) was proved in a very wide range of pH 1 to 10 for PSS, which can avoid pH adjustment in real applications. The adsorption is very fast and reaches equilibrium within 120 and 10 minutes for TR and PSS, respectively. The kinetics of Cr(VI) removal by the adsorbents was well expressed by the pseudo-second-order model. The predicted adsorption capacities of TR and PSS were found to be 15.6 mg/g and 8.5 mg/g, respectively, confirming the effectiveness of the adsorbents for Cr(VI) ions removal from water/wastewater. The possibility of reusing adsorbents up to the 3^rd^ and 4^th^ cycles for TR and PSS, respectively, indicates the potential applicability of TR and PSS for Cr(VI) from water. Besides, it verifies the valorization of biowastes for water/wastewater treatments. Further study on evaluation of the efficiency of adsorbents using a real chromium contaminated wastewater sample, in batch and column modes, is required to scale up the process for real applications.

## Figures and Tables

**Table 1:** Physiochemical properties of adsorbents.

Parameter	TR	PSS
Moisture content (%)	6.7	10.0
Dry matter (%)	93.3	90.0
Ash (%)	14.8	14.9
Volatile matter (%)	12.0	13.2
Fixed carbon (%)	66.5	61.9
Surface area (m^2^/g)	59.3	132.6
pH	3.27	6.12

**Table 2:** Parameters of the PFO and PSO kinetic models for Cr(VI) adsorption by TR and PSS.

Parameter	TR		PSS	
	PFO	PSO	PFO	PSO
Co (mg/L)	5	5	5	5
*q*_e,exp_ (mg/g)	2.0224	2.0224	2.1980	2.1980
*q*_e,cal_ (mg/g)	1.958	2.0635	2.1876	2.2044
*k*_1_ (min^−1^)	3.979	—	3.010	—
*k*_2_ (g/(mg·min))	—	3.349	—	7.358
*V*_0_ (mg/(g·min))	—	14.26	—	35.76
*R* ^2^	0.8774	0.9710	0.5457	0.8727
*χ* ^2^	0.64509	0.03468	0.27742	0.00541

**Table 3:** Isotherm parameters of the adsorption of Cr(VI) on TR and PSS.

Isotherm	Parameters	TR	PSS
Langmuir	*q*_max_ (mg/g)	15.61	8.45
	*b* (L/mg)	0.2867	33.0795
	*R*_L_	0.0526–0.7665	0.0005–0.0277
	*χ* ^2^	1.40346	9.86768
	*R* ^2^	0.9507	0.8851
Freundlich	*K*_F_ ((mg^1−1/*n*^L^1/*n*^)/g)	4.490	6.141
	*n*	2.729	8.166
	*χ* ^2^	0.3283	0.90006
	*R* ^2^	0.9885	0.9529

**Table 4:** Comparison of maximum adsorption capacities of Cr(VI) in various adsorbents with and without activation.

Adsorbent	pH	*Q*_m_ (mg/g)	Dose (g/L)	Reference
Activated carbon of *Leucaena leucocephala*	4	13.85	6	[32]
Compost	2	6.25	10	[41]
Sunflower stem	2	5.8	4	[47]
Potato powder peels	2.5	3.28	4	[48]
Untreated papaya peel	1	7.16	16.7	[46]
Wheat brane	3	0.942	20	[49]
Paper mill sludge	4	23.18	3.5	[50]
Walnut shell	3.5	8.01	10	[51]
Hazelnut shell	3.5	8.28	10	[51]
Almond shell	3.5	3.40	10	[51]
Almond green hull	2	10.12	2	[21]
TR	2	15.6	2.5	This study
PSS	3	8.5	2.5	This study

## Data Availability

The datasets used and/or analyzed during the current study are available from the corresponding author on reasonable request.
